# Effects of bilateral sequential theta-burst stimulation on 5-HT_1A_ receptors in the dorsolateral prefrontal cortex in treatment-resistant depression: a proof-of-concept trial

**DOI:** 10.1038/s41398-023-02319-3

**Published:** 2023-02-01

**Authors:** Matej Murgaš, Jakob Unterholzner, Peter Stöhrmann, Cécile Philippe, Godber M. Godbersen, Lukas Nics, Murray B. Reed, Chrysoula Vraka, Thomas Vanicek, Wolfgang Wadsak, Georg S. Kranz, Andreas Hahn, Markus Mitterhauser, Marcus Hacker, Siegfried Kasper, Rupert Lanzenberger, Pia Baldinger-Melich

**Affiliations:** 1 Department of Psychiatry and Psychotherapy, Clinical Division of General Psychiatry, Medical University of Vienna, Vienna, Austria; 2 Comprehensive Center for Clinical Neurosciences and Mental Health, Medical University of Vienna, Vienna, Austria; 3 Department of Biomedical Imaging and Image-guided Therapy, Division of Nuclear Medicine, Medical University of Vienna, Vienna, Austria; 4 Department of Rehabilitation Sciences, The Hong Kong Polytechnic University, Hung Hom, Hong Kong; 5 Ludwig Boltzmann Institute Applied Diagnostics, Vienna, Austria; 6 Department of Chemistry, Institute of Inorganic Chemistry, University of Vienna, Vienna, Austria

**Keywords:** Depression, Neuroscience

## Abstract

Theta-burst stimulation (TBS) represents a brain stimulation technique effective for treatment-resistant depression (TRD) as underlined by meta-analyses. While the methodology undergoes constant refinement, bilateral stimulation of the dorsolateral prefrontal cortex (DLPFC) appears promising to restore left DLPFC hypoactivity and right hyperactivity found in depression. The post-synaptic inhibitory serotonin-1A (5-HT_1A_) receptor, also occurring in the DLPFC, might be involved in this mechanism of action. To test this hypothesis, we performed PET-imaging using the tracer [*carbonyl*-^11^C]WAY-100635 including arterial blood sampling before and after a three-week treatment with TBS in 11 TRD patients compared to sham stimulation (*n* = 8 and *n* = 3, respectively). Treatment groups were randomly assigned, and TBS protocol consisted of excitatory intermittent TBS to the left and inhibitory continuous TBS to the right DLPFC. A linear mixed model including group, hemisphere, time, and Hamilton Rating Scale for Depression (HAMD) score revealed a 3-way interaction effect of group, time, and HAMD on specific distribution volume (V_S_) of 5-HT_1A_ receptor. While post-hoc comparisons showed no significant changes of 5-HT_1A_ receptor V_S_ in either group, higher 5-HT_1A_ receptor V_S_ after treatment correlated with greater difference in HAMD (r = −0.62). The results of this proof-of-concept trial hint towards potential effects of TBS on the distribution of the 5-HT_1A_ receptor. Due to the small sample size, all results must, however, be regarded with caution.

## Introduction

Major depressive disorder (MDD) is a common, debilitating psychiatric illness that – along with personal suffering and psychosocial strain – represents an immense socioeconomic burden worldwide [1, 2]. MDD is a treatable disorder with pharmacological and psychotherapeutic interventions constituting the fundamental pillars of antidepressant treatment. Nevertheless, up to 60% of the patients do not satisfactorily respond to first-line pharmacological treatments [3]: This subgroup of patients is conventionally labeled as treatment-resistant, which implies the failure of response to at least two adequate antidepressant trials [4]. The significant amount of affected persons calls for an improvement of treatment outcomes by exploring alternative treatment strategies, such as novel rapid-acting antidepressants like esketamine and improved brain stimulation techniques [5–7].

Theta-burst stimulation (TBS), an enhanced derivative of repetitive transcranial magnetic stimulation (rTMS), is an effective non-pharmacological treatment option for MDD, combining the approved efficacy of rTMS while offering better practicability with significantly shorter therapy duration [8, 9]. Several double-blind, sham-controlled, multicenter trials as well as meta-analyses have provided evidence for the antidepressant effects of rTMS [10, 11], which has been approved by the United States Food and Drug Administration (FDA) as a therapeutic option for treatment-resistant depression (TRD) since 2008 [12, 13]. Since the development of this noninvasive brain stimulation technique in the 1980s [14], research into therapeutic TMS for a multitude of neurological and psychiatric disorders, particularly depression [15], has dramatically increased; its principle lies in the induction of electrical currents inside the brain by electromagnetic pulses applied on the scalp, causing neuronal depolarization and functional alteration of brain activity in specifically targeted regions.

A key variable in TMS is the application frequency of electromagnetic pulses. While low frequency or single pulses were shown to decrease brain activity in the stimulated region [16], high-frequency pulses exhibit excitatory properties e.g., the FDA-approved 10-Hz or high-frequency (HF) TMS over the left dorsolateral prefrontal cortex (DLFPC) in depression [17]. Similarly, two protocols of TBS with opposing effects on cortical excitability have been proposed; intermittent (iTBS) and continuous TBS (cTBS) with excitatory and inhibitory consequences, respectively. Bilateral TBS, which combines iTBS to the left, alleged hypoactive DLPFC and cTBS to the right, alleged hyperactive DLPFC holds promise to be the most efficacious neuromodulation measure in TRD [18, 19].

Though the exact neurobiological mechanisms by which TMS alters mood remain to be elucidated, the commonly acknowledged explanation involves therapeutic neuroplasticity through long-lasting modulation of cortical excitability that go beyond the stimulated brain region [20–22]. To investigate the TMS-induced changes in neural activity patterns, several neuroimaging studies have been performed to identify brain networks involved in its antidepressant effects and inform personalized approaches in the future [23–27]. The vast majority of the trials using positron emission tomography (PET) focused on measures of cerebral blood flow [28–31] and cerebral glucose metabolism [32], showing changes in neural network dynamics beyond the cortical site directly targeted by the electromagnetic pulse. Regarding effects on modulatory neurotransmitter systems, rTMS was shown to be accompanied by increases in extracellular dopamine in the stimulated hemisphere (basal ganglia, anterior cingulate and medial orbitofrontal cortex) measured using [^11^C]raclopride [33–35], significant changes in regional serotonin synthesis capacity in limbic areas assessed using alpha-[^11^C]-methyltryptophan [36] as well as a serum serotonin level enhancement [37]. Preclinical investigations in rats indicate that TMS might exert its antidepressant effects via modulation of the serotonergic system [38, 39], particularly the inhibitory serotonin-1A (5-HT_1A_) [39–42]. This receptor is prone to profound changes in mood and anxiety disorders [43–46] and represents an important player of antidepressant pharmacotherapy and electroconvulsive therapy in humans as shown previously by our group [47–50]. Based on this evidence, we aimed at assessing the impact of bilateral TBS on 5-HT_1A_ receptor distribution in a sample of TRD patients using the radioligand [*carbonyl*-^11^C]WAY-100635 to probe the hypothesis of a 5-HT_1A_ receptor reduction in the DLFPC – as seen with other antidepressant treatments – by this non-invasive brain stimulation technique in vivo.

## Material and methods

### Subjects and study design

35 subjects suffering from treatment-resistant depression (defined as failure to respond for the current episode to two adequate medication trials of at least 4 weeks in sufficient dosage) were recruited via the outpatient department and the hospital wards of the Department of Psychiatry and Psychotherapy at the Medical University of Vienna, Austria, and enrolled in the study (ClinicalTrials.gov Identifier NCT02810717). 24 subjects dropped out of the study, mainly due to technical and schedule planning issues, leaving a final sample size of 11. In this randomized and double-blind clinical trial patients received either bilateral theta-burst (*n* = 8) or sham (*n* = 3) stimulation. Each participant underwent PET measurement with [*carbonyl*-^11^C]WAY-100635 once before (PET1) and once after TBS treatment (PET2). In addition, structural images were recorded using magnetic resonance imaging (MRI) scans at each PET scanning session, which were used for neuro-navigation and co-registration of dynamic PET data.

Subjects were carefully screened by a psychiatrist and included in the trial when fulfilling criteria for a single or recurrent major depression (using the Structural Clinical Interview for DSM IV Diagnosis, SCID IV) and a 17-item Hamilton Rating Scale for Depression (HAMD) score ≥18 (at least moderate depression) assessed at the inclusion as well as on the individual measurement days. Concomitant antidepressant treatment was allowed, if stable, four weeks prior study enrollment und during study participation. Exclusion criteria were major systemic (untreated) or neurological disorders, including brain injuries, current substance abuse (ruled out using SCID IV and a urine drug screening), current psychotic symptoms, pregnancy and any contraindication for magnetic resonance imaging or TMS [51]. Also, intake within four weeks prior the first examination visit or current intake of psychotropic drugs targeting the 5-HT_1A_ receptor (i.e. clozapine, aripiprazole, quetiapine (>100 mg), ziprasidone, amitriptyline, nebivolol, propranolol, mirtazapine, triptans, trazodone) was considered as an exclusion criterion. A causal relationship of mood disturbances and general medical conditions was further ruled out by clinical examination, routine laboratory measurements (complete blood cell count, chemistry, thyroid hormones) and an electrocardiogram.

Study data were collected and managed using REDCap electronic data capture tools hosted at the Department of Psychiatry and Psychotherapy, Medical University of Vienna, Austria [52, 53]. The study was approved by the ethics committee of the Medical University of Vienna, Austria (1761/2015). Each subject provided written informed consent and was financially reimbursed for the participation in the study. The authors assert that all procedures contributing to this work comply with the ethical standards of the relevant national and institutional committees on human experimentation and with the Helsinki Declaration of 1975, as revised in 2008.

### Theta-burst stimulation treatment

Patients were treated with bilateral TBS or sham TBS using MagPro X100 model (MagVenture, Tonica Elektronik A/S, Denmark, www.tonika.dk) and a Cool-B70 Butterfly coil. Patients were assigned to one of the two treatment arms using a computer-generated random allocation created using R (RStudio, Inc.). For each treatment session intermittent TBS (iTBS) was applied to the left dorsolateral prefrontal cortex (DLPFC), whereas continuous TBS (cTBS) was applied to the right DLPFC at an intensity of 120% resting motor threshold for the first dorsal interosseous muscle [54]. The iTBS consisted of 2-second trains with an inter-train-interval of 8 s. Trains (30 pulses, 10 bursts) were repeated 20 times to reach a total number of 600 pulses per session. The cTBS comprised uninterrupted bursts reaching a total number of 600 pulses per session. For both iTBS and cTBS 3-pulse 50-Hz bursts were given every 200 ms [9]. Two sessions, each lasting ~5 min, were scheduled daily, given 60 min apart [55], Monday to Friday, for 3 weeks resulting in a minimum of 30 sessions per subjects. Coil placement to the left and right DLPFC was performed using neuro-navigation (Brainsight, LOCALITE® TMS Navigator, Germany [56]) based on MNI coordinates *x* = ± 38, *y* = 44, *z* = 26 from individual MR images. Sham TBS comprised bursts as given above with the coil set at 90° against the skull. Thus, sham stimulation was accompanied by similar auditory (clicking noise) and somatosensory (i.e. pricking) artefacts. Patients were blind to the individual group assignment. Efficacy outcome measures were assessed by blinded raters, who were not permitted access to the treatment sessions. Un-blinding of both patients and raters happened after the second PET measurement.

### Neuroimaging

Each PET scan was conducted using a GE Advance PET scanner (General Electric Medical Systems, Milwaukee, Wisconsin) at the Department of Biomedical Imaging and Image-guided Therapy, Division of Nuclear Medicine, Medical University of Vienna, Austria as previously described [49, 50, 57, 58]. To correct for tissue attenuation, 5-min transmission scan was carried in 2-D mode (retractable ^68^Ge rod sources). Afterwards, PET measurement started with the bolus administration of [*carbonyl*-^11^C]WAY-100635 (injection dose 4.6 MBq/kg body weight) in cubital vein. All scans were acquired in 3-D mode for 90 min (51 frames: 12 × 5 s, 6 × 10 s, 3 × 20 s, 6 × 30 s, 9 × 60 s, 15 × 300 s) and were reconstructed (iterative filtered back-projection algorithm) to final images comprising a spatial resolution of 4.36 mm full-width at half-maximum 1 cm next to the center of the field of view (matrix 128 × 128, 35 slices). The radioligand [*carbonyl*-^11^C]WAY-100635 was prepared according to previously published methods [59] at the Cyclotron Unit of the PET Center.

Each PET scan was complemented with arterial blood samples for the quantification of [*carbonyl*-^11^C]WAY-100635 that were automatically drawn for first 10 min (ALLOGG, Mariefred, Sweden) and manually at 2, 5, 6, 7, 8, 10, 20, 40, and 60 min of the measurement.

Structural T1-weighted MR image were acquired at both PET measurements with the magnetization prepared rapid gradient echo sequence (MP-RAGE with TE/TR = 4.21/3000 ms, voxel size 1 × 1 × 1.1 mm^3^) using a 3 T PRISMA MR Scanner (Siemens Medical, Erlangen, Germany).

### Data processing and quantification

Following correction for tissue attenuation, PET scan of each patient was corrected for head motion, co-registered to the structural T1-weighted image. The latter was afterwards normalized to the Montreal Neurological Institute (MNI) space producing a transformation matrix that was further applied to normalize co-registered PET data to MNI space. All preprocessing steps were done using SPM (Wellcome Trust Centre for Neuroimaging, London, United Kingdom; http://www.fil.ion.ucl. ac.uk/spm/) and Matlab 2018a (The Mathworks Inc., Natick, MA, USA). Subsequently, time activity curves (TACs) were extracted for selected regions of interest (ROIs) - left and right DLPFC and cerebellar white matter (CWM). DLPFC ROIs were defined as a sphere with diameter of 10 mm around the MNI coordinate representing the individual application point of TBS treatment. The CWM ROI was extracted using an in-house created atlas [60]. To reduce the noise induced by short frames in the beginning of the scan, the first 2 min (frames 12 × 5 s and 6 × 10 s) of the measurement were resampled to 20-s frames.

The arterial input functions representing non-metabolized radioligand in plasma were obtained as product of the whole blood activity, plasma-to-whole blood ratio (average) and fraction of intact radioligand in the plasma (fitted with the Hill-type function). Afterwards, the specific volume of distribution (V_S_), representing the amount of radioligand bound solely to the target 5-HT_1A_ receptor in the investigated target tissue, i.e. in DLPFC. Here, distribution volume V_S_ is equal to the binding potential (BP_P_) of 5-HT_1A_ receptor as defined by Innis et al. 2007 [61]. Quantification of 5-HT_1A_ receptor V_S_ was carried out utilizing a constrained two-tissue compartment model. Here, CWM was fitted and the ratio of K_1_/k_2_ (K_1_ - rate constant for transfer from arterial plasma to tissue, k_2_ - rate constant for transfer from tissue to arterial plasma) was fixed for the DLPFC regions [62]. Model fitting and quantification of [*carbonyl*-^11^C]WAY-100635 was carried out in PMOD 4.201 (PMOD Technologies Ltd., Zurich, Switzerland; www.pmod.com).

### Statistical analysis

All statistical analyses were performed in SPSS version 28 for Windows (SPSS Inc., Chicago, Illinois, USA; www.spss.com). A linear mixed model was used to assess the effect of TBS treatment on 5-HT_1A_ receptor V_S_ in the DLPFC in TRD patients using group assignment (TBS or sham), time point of measurement (PET1 or PET2) and hemisphere of ROI as fixed factors and HAMD scores, representing a scale predictor, as covariate. Of note, the factor hemisphere was introduced in the statistical model as iTBS and cTBS to the left and right DLPFC, respectively, are presumed to display opposing effects on brain activation, thereby potentially bearing lateralized effects on 5-HT_1A_ receptor distribution.

The Mann–Whitney U-test was utilized to assess possible difference in the baseline HAMD score between both groups. Post-hoc exploratory tests for the interactions were done using Wilcoxon Signed Ranked Test. The relationship between V_S_ and HAMD was investigated using the Spearman’s Rank correlation via change in HAMD (${\Delta}HAMD = HAMD_{PET2} - HAMD_{PET1}$) and the change in V_S_ (${\Delta}V_S = V_{S\_PET2} - V_{S\_PET1}$) for the verum group.

In addition, lateralization quotient (LQ) [63] describing the difference between the activation in left and right hemisphere was calculated for each time point (PET1 and PET2) for TBS group for DLPFC (see Table 2).

$$LQ\left[ \% \right] = \frac{{V_{{{{\mathrm{S}}}}\_{{{\mathrm{left}}}}} - V_{{{{\mathrm{S}}}}\_{{{\mathrm{right}}}}}}}{{V_{{{{\mathrm{S}}}}\_{{{\mathrm{left}}}}} + V_{{{{\mathrm{S}}}}\_{{{\mathrm{right}}}}}}} \times 100$$

Afterwards, a Wilcoxon Signed Ranks Test was used for possible changes in LQ between PET1 and PET2. All statistical tests were assessed on the significance level 0.05. No further corrections for multiple testing were done, as the analysis is of exploratory nature.

## Results

Data from eleven TRD subjects were available to examine the impact of three weeks of TBS on 5-HT_1A_ receptor V_S_ in the left and right DLPFC. The sample’s demographics are summarized in Table 1. Eight subjects (five women) aged 35.9 ± 8.4 received i/cTBS, three subjects (only women) aged 40.3 ± 8.0 received sham stimulation. Mean baseline HAMD scores were 18.5 ± 3.6 and 22.0 ± 2.7, respectively, and comparable in both groups (Mann–Whitney U test, *p* = 0.18). Concomitant medication of the participants is subsumed in the Supplementary table S1.

**Table 1:** Demographic information about the patients included in the study.

Group	TBS	Sham
*n*	8 (5 female)	3 (3 female)
Age	35.88 ± 8.41	40.33 ± 6.03
Baseline HAMD (PET1)	18.50 ± 3.55	22.00 ± 2.65
HAMD after treatment (PET2)	12.12 ± 5.44	17.33 ± 10.02
Number of responders	2	1

Response to treatment was defined as a reduction of baseline HAMD ≥ 50%. 2 out of 8 (25%) TBS-treated subjects fulfilled these criteria at PET2 (after TBS), 1 out of 3 (33%) in the sham group.

**Fig. 1:**
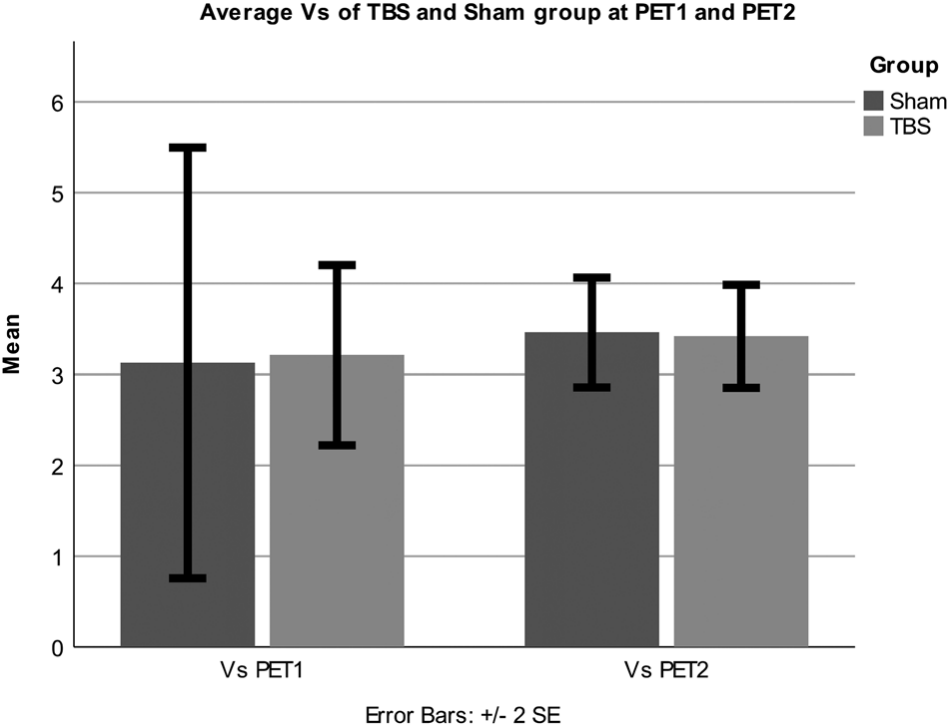
Average 5-HT_1A_ receptor *V*_S_. Bar plot showing mean (± standard error SE) of *V*_S_ in DLPFC for TBS and Sham group at both measurement time points.

Linear mixed model analysis using group (TBS vs. sham), time (PET1 vs. PET2), hemisphere (left vs. right) and HAMD score showed a main effect of group (*F* = 6.75, *p* = 0.019), time (*F* = 7.45, *p* = 0.015), and HAMD (*F* = 11.00, *p* = 0.004) on 5-HT_1A_ receptor V_S_ as well as two-way interactions between group*time (*F* = 6.24, *p* = 0.024), time*HAMD (*F* = 7.30, *p* = 0.015), group*HAMD (*F* = 6.10, *p* = 0.025), and a three-way interaction between group*time*HAMD (*F* = 6.02, *p* = 0.025). All other two- or three-way interactions and the main effect of hemisphere were non-significant. Post-hoc comparisons using Wilcoxon Signed Ranks test revealed no significant changes of 5-HT_1A_ receptor V_S_ at PET2 compared to PET1 in the TBS (*p* = 0.67) and sham group (*p* = 1.00). The estimates of 5-HT_1A_ receptor V_S_ (averaged over hemispheres) were 3.21 ± 1.40 at PET1 and 3.42 ± 0.80 at PET2 in the TBS group, and 3.13 ± 2.05 at PET1 and 3.46 ± 0.52 at PET2 in sham group (see Fig. 1).

**Fig. 2:**
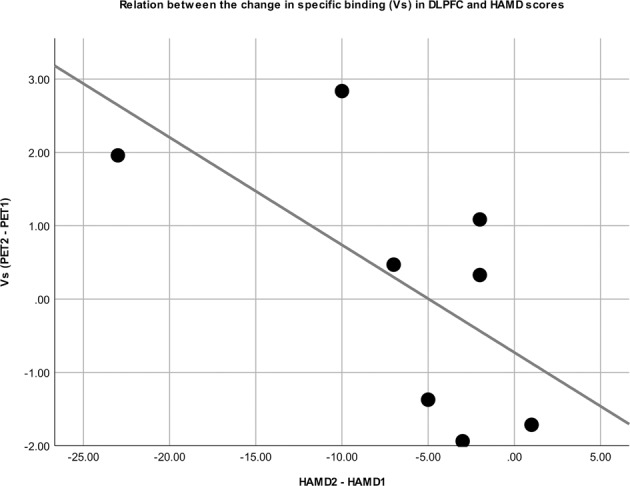
Correlation between HAMD and 5-HT_1A_ receptor *V*_s_. Scatter plot showing the relationship between the ΔHAMD score and Δ*V*_S_ between PET1 and PET2 in the TBS group. Spearman’s rank correlation showed *r* = 0.62 (*p* = 0.099).

Spearman’s rank correlation between change of 5-HT_1A_ receptor V_S_ and ΔHAMD between both PET measurements revealed a negative correlation in the TBS group (*r* = −0.62, *p* = 0.0999; see Fig. 2). Due to the small sample size, this correlation is not reported for the sham group (*n* = 3).

LQ was computed separately for each group (see Table 2) and did not change following treatment in the TBS group (Wilcoxon-Signed Rank test, *p* = 0.069). Due to the small sample size, we did not perform this test in the sham group (*n* = 3).

**Table 2:** Average lateralization quotient of V_S._ Lateralization quotient was calculated for each time point and each group separately.

Group	TBS	Sham
LQ PET1	15.58 ± 3.55	3.28 ± 6.52
LQ PET2	−2.13 ± 7.68	−6.10 ± 7.71

## Discussion

Specific distribution volumes of 5-HT_1A_ receptor in the stimulation epicenters located in the left and right DLPFC as assessed using [*carbonyl*-^11^C]WAY-100635 appeared to be differentially affected by three weeks of bilateral TBS treatment (iTBS over the left and cTBS over the right DLPFC) compared to sham stimulation in a sample of eleven TRD patients. Given the small sample size particularly in the sham group, the results of this longitudinal PET study should be considered exploratory and must therefore be interpreted carefully.

Based on earlier imaging findings published by our group using the same radioligand (see below), we expected a reduction of 5-HT_1A_ receptor V_S_ in the target region upon completion of TBS. In fact, a reduced availability of 5-HT_1A_ receptors might represent a common neural ground for pharmacological and non-pharmacological antidepressant treatments. Three months of escitalopram intake in patients with anxiety disorders were accompanied by significant reductions in binding potentials in limbic regions [48]. The same direction of change was shown in the raphe in a medication-free depressed sample after SSRI treatment [64]. Interestingly, reductions of 5-HT_1A_ receptor binding were also observed in cortical brain regions following eight weeks of daily intake of Silexan^®^, an anxiolytic herbal compound of lavender essential oil [50]. Most pronounced and widespread cortical reductions of 5-HT_1A_ receptor binding (~30%) were reported following a course of ECT [49]. However, in none of the studies mentioned above was the degree of binding reductions over time correlated with treatment outcomes. Also, contrasting results regarding 5-HT_1A_ receptor binding might arise through differences in the methodology used, including the choice of reference region and modeling [65, 66].

In the present study, though post-hoc tests did not reveal significant differences in 5-HT_1A_ receptor V_S_ between both PET scans, absolute numbers indicate a slight and unexpected increase of outcome measures in the stimulation epicenters located in the DLPFC in both groups. In addition, the Spearman’s rank correlation between the change of 5-HT_1A_ receptor V_S_ and HAMD after the treatment course suggests the greater the increase of Vs after the treatment course, the greater the reduction of HAMD scores (and the greater the response). Since we did not find significant differences between hemispheres, left iTBS and right cTBS seem to similarly affect the distribution of the 5-HT_1A_ receptor. Given the general assumption that activation of 5-HT_1A_ receptors in projection areas mediates a hyperpolarizing response to serotonin on pyramidal neurons and GABA-ergic neurons [67], an increase of 5-HT_1A_ availability in the DLPFC might result in a disinhibition of neurotransmission and increase in neuronal activity. Though highly speculative, in theory, this constitutes the desired effect of iTBS to the left DLPFC in depression [18]. Currently, no previous in vivo data exists to explain our results; however, novel cellular models might be promising to test this hypothesis [68].

Still, there is a high level of preclinical evidence supporting our hypothesis of TMS-induced changes within the serotonergic system. Several animal studies have indicated that rTMS may affect the serotonergic system through the 5-HT_1A_ receptors, the most important inhibitory receptor subtype within the serotonergic receptor family, either expressed as an autoreceptor on presynaptic serotonergic neurons or as a heteroreceptor on postsynaptic neurons in projection sites [46]. Single rapid-rate rTMS exposure led to significant increases in 5-HT_1A_ receptors in the frontal cortex as quantified by in-vitro autoradiography 24 h after the intervention in rats [39]. Chronic rTMS reduced the ability of OH-DPAT, a full 5-HT_1A_ receptor agonist, to decrease serotonin levels in projection sites, which is indicative of a reduced sensitivity of 5-HT_1A_ autoreceptors [41]. Investigations using intracerebral microdialysis indicate the selective release of monoamines following rTMS, however not necessarily of serotonin [39, 69, 70]. Only few reports show an rTMS-induced serotonin level increase in the rat hippocampus [38, 71] and nucleus accumbens [72], an effect that was also reported in humans [37]. In contrast to the 5-HT_1A_ receptor, the major excitatory serotonin receptor 5-HT_2A_ was shown to be downregulated by chronic rTMS in rats [73]; interestingly, in humans, decrease of 5-HT_2A_ receptors in the hippocampus and the bilateral DLPFC was correlated with treatment response to HF rTMS [74]. Finally, evidence retrieved from genetic investigations emphasizes the association of the 5-HT_1A_ receptor and TMS, as the genotype of the 5-HT_1A_ receptor promoter region polymorphism (rs6295) was shown to influence the outcome of HF TMS in patients suffering from a major depressive episode [75, 76]. It has been suggested that a greater load of G alleles in a 5-HT_1A_ receptor promotor polymorphism might be associated with lower serotonin release, resulting in a post-synaptic upregulation of the 5-HT_1A_ receptor [65]. This polymorphism was, however, not assessed in our study sample. Recently, a PET study in dogs showed a reduction of serotonin transporter binding and therefore availability in the subgenual anterior cingulate cortex (sgACC) one month after a four-day accelerated rTMS stimulation protocol of the prefrontal cortex [77]. The study thus highlights the importance of the serotonergic system in the mechanisms of TMS and might hint towards an involvement of the 5-HT_1A_ receptor, since the sgACC and the DLPFC are both 5-HT_1A_ receptor rich regions and share dense, reciprocal connections [78, 79].

The effect of TMS in TRD is also frequently associated with plastic changes affecting synapse formation, long-term potentiation (LTP) and depression (LTD) [80, 81]. In a study in mice, Cambiaghi et al. have shown, for example, that high-frequency rTMS leads to an increase in dendritic complexity in layer II/III pyramidal neurons of the primary motor cortex [82]. The 5-HT_1A_ receptor, in combination with 5-HT signaling, has been repeatedly implicated in plastic changes, including alterations in gray matter volumes [83, 84]. Since the receptor is also located on pyramidal neurons in layers III [85], rTMS might influence its expression and thus mediate its effect on synaptogenesis. Of note, rTMS of glial cells affects neuronal excitability and might, through the presence of 5-HT_1A_ receptors on glial cells [86], lead to alterations in 5-HT_1A_ availability and thus contribute to neuroplasticity [87]. Data on the interplay of rTMS and 5-HT_1A_ receptors in pyramidal and glial cells in cell cultures is however missing up until now [86].

According to the chosen treatment protocol in this study, applying (excitatory) iTBS to the left DLPFC and (inhibiting) cTBS to the right DLPFC, we would have expected a clearer change in LQ between PET1 and PET2 in the TBS group. However, the latter was not significant. Considering the absolute values of the computed index, the LQ was positive at baseline in both groups, implying a higher 5-HT_1A_ receptor binding on the left compared to the right hemisphere in symptomatic, depressed patients, and negative following three weeks of treatment, corresponding to a 5-HT_1A_ distribution reversal that seems more pronounced in the TBS group. This is in accordance with the presumed left and right hemispheric divergence of metabolism and neuronal activation in depression [88, 89] suggesting that our results might have shown clearer trends in the TBS group in a larger sample [90].

Regarding the clinical data of our population, the HAMD scores at baseline in both the verum and sham groups were somewhat lower than in other studies investigating TMS in TRD [55, 91, 92], but are still reflective of an at least moderate depressive episode. The response rates were 25% in the verum group (2 out of 8), and 33.3% in the sham group (1 out of 3). While these rates seem to differ from recent studies by Berlim et al. and Voigt et al. [19, 93], a meta-analysis of Lepping et al. also finds high sham response rates [94]. The technology of transcranial magnetic stimulation undergoes constant refinement, hence studies on effects of TMS show great heterogeneity in treatment protocols (and duration) and inclusion criteria. Of note, in the current study recruitment was limited to patients currently not receiving treatment with mirtazapine, trazodone, quetiapine, aripiprazole, compatible for PET imaging of the 5-HT_1A_ receptor, as well as patients not receiving antiepileptic drugs or benzodiazepines on a regular basis regarding TBS treatment. Also, trajectories of remission and response to TMS seem to depend on specific characteristics, including age, benzodiazepine use and baseline depression severity [95]. An adequate level of functioning represents one condition for TBS, particularly in an outpatient setting, allowing for the inclusion of less severely depressed TRD patients (in comparison to, for example electroconvulsive therapy [96]). In addition, daily sessions might provide for a certain level of activation that could influence depression scores and symptom improvement over the treatment course. While treatment protocols for TBS are continuously refined based on new evidence, the herein reported response rates must be considered with caution, especially with our comparatively small sham group.

The size of the sham group (comprising female subjects only), but also the general sample size of 11 subjects constitutes the most important limitation of this study, restricting the generalizability of our data [97]. Screening and recruitment were performed based on previous power considerations to observe effects of iTBS on symptom reduction and group differences in PET data [49, 55, 98]. Bearing in mind the specific inclusion and exclusion criteria (including TRD and PET imaging), 35 subjects could be enrolled before the study had to be terminated early. Data of 24 of these 35 subjects could not be used since they had to be dropped out due to technical, medical, and personal reasons, including reconstruction errors, missing arterial input functions or withdrawal of consent for PET measurements. The dropouts were necessary for quality control, to maintain the gold-standard of PET analysis and reduce data variability [99]. To increase statistical sensitivity, we focused our analysis on the bilateral DLPFCs where TBS was administered. However, other ROIs, especially the raphe, but also the sgACC, the hippocampus and amygdala, where changes in 5-HT_1A_ receptor might be expected, are missing in this analysis. Also, we did not account for potential effects of concomitant antidepressant pharmacotherapy in our analyses [100]. All patients had taken at least two antidepressive medications in sufficient duration and dosage before inclusion and currently received one or more agents acting on the serotonergic system (see Supplementary Table 1). Different classes of antidepressant medication affect the functioning of 5-HT_1A_ receptors. SSRIs, MAO inhibitors, α_2_-antagonists and electric shocks seem to increase tonic activation of postsynaptic serotonin-1_A_ receptors in the hippocampus [101]. However, the extent varies depending on the substance and the brain region [102]. Also, the effects on 5-HT_1A_ functioning seem to be independent of changes in receptor density [99]. We therefore expect the effects of the concomitant medication to be negligible especially since treatment regimens had been stable for at least for weeks and had to remain unchanged throughout study participation.

In conclusion, we could show an effect of three-week bilateral TBS treatment on the distribution volumes of the 5-HT_1A_ receptor in a group of patients suffering from treatment-resistant depression. While these results appear indicative of a connection of the 5-HT_1A_ receptor with the mechanisms of action of theta-burst stimulation, they must be interpreted with caution, particularly because of the small sample and sham group size.

## Supplementary information

Supplemental material

## References

[1] Rehm J, Shield KD (2019). Global burden of disease and the impact of mental and addictive disorders. Curr Psychiatry Rep.

[2] Smith K (2011). Trillion-dollar brain drain. Nature.

[3] Bartova L, Dold M, Kautzky A, Fabbri C, Spies M, Serretti A (2019). Results of the European Group for the Study of Resistant Depression (GSRD) - basis for further research and clinical practice. World J Biol Psychiatry.

[4] Dold M, Kasper S (2017). Evidence-based pharmacotherapy of treatment-resistant unipolar depression. Int J Psychiatry Clin Pract.

[5] Kraus C, Kadriu B, Lanzenberger R, Zarate CA, Kasper S (2019). Prognosis and improved outcomes in major depression: a review. Transl Psychiatry.

[6] Kasper S, Cubała WJ, Fagiolini A, Ramos-Quiroga JA, Souery D, Young AH (2021). Practical recommendations for the management of treatment-resistant depression with esketamine nasal spray therapy: Basic science, evidence-based knowledge and expert guidance. World J Biol Psychiatry.

[7] Blumberger DM, Mulsant BH, Daskalakis ZJ (2013). What is the role of brain stimulation therapies in the treatment of depression?. Curr Psychiatry Rep.

[8] Blumberger DM, Vila-Rodriguez F, Thorpe KE, Feffer K, Noda Y, Giacobbe P (2018). Effectiveness of theta burst versus high-frequency repetitive transcranial magnetic stimulation in patients with depression (THREE-D): a randomised non-inferiority trial. Lancet.

[9] Huang YZ, Edwards MJ, Rounis E, Bhatia KP, Rothwell JC (2005). Theta burst stimulation of the human motor cortex. Neuron.

[10] Lefaucheur JP, Aleman A, Baeken C, Benninger DH, Brunelin J, Di Lazzaro V (2020). Evidence-based guidelines on the therapeutic use of repetitive transcranial magnetic stimulation (rTMS): An update (2014–2018). Clin Neurophysiol.

[11] Berlim MT, van den Eynde F, Tovar-Perdomo S, Daskalakis ZJ (2014). Response, remission and drop-out rates following high-frequency repetitive transcranial magnetic stimulation (rTMS) for treating major depression: a systematic review and meta-analysis of randomized, double-blind and sham-controlled trials. Psychological Med.

[12] O’Reardon JP, Solvason HB, Janicak PG, Sampson S, Isenberg KE, Nahas Z (2007). Efficacy and safety of transcranial magnetic stimulation in the acute treatment of major depression: a multisite randomized controlled trial. Biol Psychiatry.

[13] George MS, Lisanby SH, Avery D, McDonald WM, Durkalski V, Pavlicova M (2010). Daily left prefrontal transcranial magnetic stimulation therapy for major depressive disorder: a sham-controlled randomized trial. Arch Gen Psychiatry.

[14] Barker AT, Jalinous R, Freeston IL (1985). Non-invasive magnetic stimulation of human motor cortex. Lancet.

[15] Höflich G, Kasper S, Hufnagel A, Ruhrmann S, Möller H-J (1993). Application of transcranial magnetic stimulation in treatment of drug-resistant major depression—a report of two cases. Hum Psychopharmacol.

[16] Casula EP, Tarantino V, Basso D, Arcara G, Marino G, Toffolo GM (2014). Low-frequency rTMS inhibitory effects in the primary motor cortex: Insights from TMS-evoked potentials. NeuroImage.

[17] Lefaucheur JP, André-Obadia N, Antal A, Ayache SS, Baeken C, Benninger DH (2014). Evidence-based guidelines on the therapeutic use of repetitive transcranial magnetic stimulation (rTMS). Clin Neurophysiol.

[18] Li H, Cui L, Li J, Liu Y, Chen Y (2021). Comparative efficacy and acceptability of neuromodulation procedures in the treatment of treatment-resistant depression: a network meta-analysis of randomized controlled trials. J Affect Disord.

[19] Berlim MT, McGirr A, Rodrigues Dos Santos N, Tremblay S, Martins R (2017). Efficacy of theta burst stimulation (TBS) for major depression: an exploratory meta-analysis of randomized and sham-controlled trials. J Psychiatr Res.

[20] Lazzaro VD, Ziemann U, Lemon RN (2008). State of the art: physiology of transcranial motor cortex stimulation. Brain Stimulation.

[21] Ilić TV, Ziemann U (2005). Exploring motor cortical plasticity using transcranial magnetic stimulation in humans. Ann N Y Acad Sci.

[22] Ziemann U, Ilić TV, Jung P (2006). Long-term potentiation (LTP)-like plasticity and learning in human motor cortex-investigations with transcranial magnetic stimulation (TMS). Suppl Clin Neurophysiol.

[23] Reithler J, Peters JC, Sack AT (2011). Multimodal transcranial magnetic stimulation: using concurrent neuroimaging to reveal the neural network dynamics of noninvasive brain stimulation. Prog Neurobiol.

[24] Garnaat SL, Fukuda AM, Yuan S, Carpenter LL (2019). Identification of clinical features and biomarkers that may inform a personalized approach to rTMS for depression. Personalized Med Psychiatry.

[25] Hernández-Ribas R, Deus J, Pujol J, Segalàs C, Vallejo J, Menchón JM (2013). Identifying brain imaging correlates of clinical response to repetitive transcranial magnetic stimulation (rTMS) in major depression. Brain Stimul.

[26] Fidalgo TM, Morales-Quezada JL, Muzy GS, Chiavetta NM, Mendonca ME, Santana MV (2014). Biological markers in noninvasive brain stimulation trials in major depressive disorder: a systematic review. J ECT.

[27] Stöhrmann P, Godbersen GM, Reed MB, Unterholzner J, Klöbl M, Baldinger-Melich P, et al. Effects of bilateral sequential theta-burst stimulation on functional connectivity in treatment-resistant depression: first results. J Affect. Disord. 2023;324:660–669. 10.1016/j.jad.2022.12.088 36603604

[28] Paus T, Castro-Alamancos MA, Petrides M (2001). Cortico-cortical connectivity of the human mid-dorsolateral frontal cortex and its modulation by repetitive transcranial magnetic stimulation. Eur J Neurosci.

[29] Knoch D, Treyer V, Regard M, Müri RM, Buck A, Weber B (2006). Lateralized and frequency-dependent effects of prefrontal rTMS on regional cerebral blood flow. NeuroImage.

[30] Speer AM, Willis MW, Herscovitch P, Daube-Witherspoon M, Shelton JR, Benson BE (2003). Intensity-dependent regional cerebral blood flow during 1-Hz repetitive transcranial magnetic stimulation (rTMS) in healthy volunteers studied with H215O positron emission tomography: II. Effects of prefrontal cortex rTMS. Biol Psychiatry.

[31] Ferrarelli F, Haraldsson HM, Barnhart TE, Roberts AD, Oakes TR, Massimini M (2004). A [17F]-fluoromethane PET/TMS study of effective connectivity. Brain Res Bull.

[32] Kimbrell TA, Dunn RT, George MS, Danielson AL, Willis MW, Repella JD (2002). Left prefrontal-repetitive transcranial magnetic stimulation (rTMS) and regional cerebral glucose metabolism in normal volunteers. Psychiatry Res.

[33] Ko JH, Monchi O, Ptito A, Bloomfield P, Houle S, Strafella AP (2008). Theta burst stimulation-induced inhibition of dorsolateral prefrontal cortex reveals hemispheric asymmetry in striatal dopamine release during a set-shifting task: a TMS-[(11)C]raclopride PET study. Eur J Neurosci.

[34] Strafella AP, Paus T, Fraraccio M, Dagher A (2003). Striatal dopamine release induced by repetitive transcranial magnetic stimulation of the human motor cortex. Brain.

[35] Strafella AP, Paus T, Barrett J, Dagher A (2001). Repetitive transcranial magnetic stimulation of the human prefrontal cortex induces dopamine release in the caudate nucleus. J Neurosci.

[36] Sibon I, Strafella AP, Gravel P, Ko JH, Booij L, Soucy JP (2007). Acute prefrontal cortex TMS in healthy volunteers: effects on brain 11C-alphaMtrp trapping. NeuroImage.

[37] Lu R, Zhang C, Liu Y, Wang L, Chen X, Zhou X (2018). The effect of bilateral low-frequency rTMS over dorsolateral prefrontal cortex on serum brain-derived neurotropic factor and serotonin in patients with generalized anxiety disorder. Neurosci Lett.

[38] Ben-Shachar D, Belmaker RH, Grisaru N, Klein E (1997). Transcranial magnetic stimulation induces alterations in brain monoamines. J Neural Transm.

[39] Kole MH, Fuchs E, Ziemann U, Paulus W, Ebert U (1999). Changes in 5-HT1A and NMDA binding sites by a single rapid transcranial magnetic stimulation procedure in rats. Brain Res.

[40] Pollandt S, Drephal C, Albrecht D (2003). 8-OH-DPAT suppresses the induction of LTP in brain slices of the rat lateral amygdala. Neuroreport.

[41] Gur E, Lerer B, Dremencov E, Newman ME (2000). Chronic repetitive transcranial magnetic stimulation induces subsensitivity of presynaptic serotonergic autoreceptor activity in rat brain. Neuroreport.

[42] Johnson MT, McCullough J, Nindl G, Chamberlain JK (2003). Autoradiographic evaluation of electromagnetic field effects on serotonin (5HT1A) receptors in rat brain. Biomed Sci Instrum.

[43] Lanzenberger RR, Mitterhauser M, Spindelegger C, Wadsak W, Klein N, Mien LK (2007). Reduced serotonin-1A receptor binding in social anxiety disorder. Biol Psychiatry.

[44] Nugent AC, Bain EE, Carlson PJ, Neumeister A, Bonne O, Carson RE (2013). Reduced post-synaptic serotonin type 1A receptor binding in bipolar depression. Eur Neuropsychopharmacol.

[45] Neumeister A, Bain E, Nugent AC, Carson RE, Bonne O, Luckenbaugh DA (2004). Reduced serotonin type 1A receptor binding in panic disorder. J Neurosci.

[46] Savitz J, Lucki I, Drevets WC (2009). 5-HT(1A) receptor function in major depressive disorder. Prog Neurobiol.

[47] Lanzenberger R, Baldinger P, Hahn A, Ungersboeck J, Mitterhauser M, Winkler D (2013). Impact of electroconvulsive therapy on 5-HT1A receptor binding in major depression. Mol Psychiatry.

[48] Spindelegger C, Lanzenberger R, Wadsak W, Mien LK, Stein P, Mitterhauser M (2009). Influence of escitalopram treatment on 5-HT 1A receptor binding in limbic regions in patients with anxiety disorders. Mol Psychiatry.

[49] Lanzenberger R, Baldinger P, Hahn A, Ungersboeck J, Mitterhauser M, Winkler D (2013). Global decrease of serotonin-1A receptor binding after electroconvulsive therapy in major depression measured by PET. Mol Psychiatry.

[50] Baldinger P, Höflich AS, Mitterhauser M, Hahn A, Rami-Mark C, Spies M (2014). Effects of Silexan on the serotonin-1A receptor and microstructure of the human brain: a randomized, placebo-controlled, double-blind, cross-over study with molecular and structural neuroimaging. Int J Neuropsychopharmacol.

[51] Rossi S, Hallett M, Rossini PM, Pascual-Leone A (2011). Screening questionnaire before TMS: an update. Clin Neurophysiol.

[52] Harris PA, Taylor R, Minor BL, Elliott V, Fernandez M, O’Neal L (2019). The REDCap consortium: building an international community of software platform partners. J Biomed Inform.

[53] Harris PA, Taylor R, Thielke R, Payne J, Gonzalez N, Conde JG (2009). Research electronic data capture (REDCap)-a metadata-driven methodology and workflow process for providing translational research informatics support. J Biomed Inform.

[54] Ge R, Blumberger DM, Downar J, Daskalakis ZJ, Dipinto AA, Tham JCW (2017). Abnormal functional connectivity within resting-state networks is related to rTMS-based therapy effects of treatment resistant depression: a pilot study. J Affect Disord.

[55] Li CT, Chen MH, Juan CH, Huang HH, Chen LF, Hsieh JC (2014). Efficacy of prefrontal theta-burst stimulation in refractory depression: a randomized sham-controlled study. Brain.

[56] Fox MD, Buckner RL, White MP, Greicius MD, Pascual-Leone A (2012). Efficacy of transcranial magnetic stimulation targets for depression is related to intrinsic functional connectivity with the subgenual cingulate. Biol Psychiatry.

[57] Hahn A, Nics L, Baldinger P, Ungersböck J, Dolliner P, Frey R. et al. Combining image-derived and venous input functions enables quantification of serotonin-1A receptors with [carbonyl-11C]WAY-100635 independent of arterial sampling. NeuroImage. 2012;62:199–206. 10.1016/j.neuroimage.2012.04.047 22579604

[58] Lanzenberger RR, Mitterhauser M, Spindelegger C, Wadsak W, Klein N, Mien L-K (2007). Reduced serotonin-1A receptor binding in social anxiety disorder. Biol Psychiatry.

[59] Wadsak W, Mien L-K, Ettlinger DE, Eidherr H, Haeusler D, Sindelar K-M (2007). 18F fluoroethylations: different strategies for the rapid translation of 11C-methylated radiotracers. Nucl Med Biol.

[60] Savli M, Bauer A, Mitterhauser M, Ding Y-S, Hahn A, Kroll T (2012). Normative database of the serotonergic system in healthy subjects using multi-tracer PET. NeuroImage.

[61] Innis RB, Cunningham VJ, Delforge J, Fujita M, Gjedde A, Gunn RN (2007). Consensus nomenclature for in vivo imaging of reversibly binding radioligands. J Cereb Blood Flow Metab.

[62] Parsey RV, Slifstein M, Hwang D-R, Abi-Dargham A, Simpson N, Mawlawi O. et al. Validation and reproducibility of measurement of 5-HT1A receptor parameters with [carbonyl-11C]WAY-100635 in humans: comparison of arterial and reference tissue input functions. J Cereb Blood Flow Metab. 2000;20:1111–33. 10.1097/00004647-200007000-00011 10908045

[63] Fink M, Wadsak W, Savli M, Stein P, Moser U, Hahn A (2009). Lateralization of the serotonin-1A receptor distribution in language areas revealed by PET. NeuroImage.

[64] Gray NA, Milak MS, DeLorenzo C, Ogden RT, Huang YY, Mann JJ (2013). Antidepressant treatment reduces serotonin-1A autoreceptor binding in major depressive disorder. Biol Psychiatry.

[65] Parsey RV, Ogden RT, Miller JM, Tin A, Hesselgrave N, Goldstein E (2010). Higher serotonin 1A binding in a second major depression cohort: modeling and reference region considerations. Biol Psychiatry.

[66] Hahn A, Nics L, Baldinger P, Wadsak W, Savli M, Kraus C.et al. Application of image-derived and venous input functions in major depression using [carbonyl-(11)C]WAY-100635. Nucl Med Biol. 2013;40:371–7. 10.1016/j.nucmedbio.2012.12.011 23375480

[67] Garcia-Garcia AL, Newman-Tancredi A, Leonardo ED (2014). 5-HT(1A) [corrected] receptors in mood and anxiety: recent insights into autoreceptor versus heteroreceptor function. Psychopharmacology.

[68] Aberra AS, Wang B, Grill WM, Peterchev AV (2020). Simulation of transcranial magnetic stimulation in head model with morphologically-realistic cortical neurons. Brain Stimul.

[69] Keck ME, Sillaber I, Ebner K, Welt T, Toschi N, Kaehler ST (2000). Acute transcranial magnetic stimulation of frontal brain regions selectively modulates the release of vasopressin, biogenic amines and amino acids in the rat brain. Eur J Neurosci.

[70] Kanno M, Matsumoto M, Togashi H, Yoshioka M, Mano Y (2004). Effects of acute repetitive transcranial magnetic stimulation on dopamine release in the rat dorsolateral striatum. J neurological Sci.

[71] Juckel G, Mendlin A, Jacobs BL (1999). Electrical stimulation of rat medial prefrontal cortex enhances forebrain serotonin output: implications for electroconvulsive therapy and transcranial magnetic stimulation in depression. Neuropsychopharmacology.

[72] Löffler S, Gasca F, Richter L, Leipscher U, Trillenberg P, Moser A (2012). The effect of repetitive transcranial magnetic stimulation on monoamine outflow in the nucleus accumbens shell in freely moving rats. Neuropharmacology.

[73] Ben-Shachar D, Gazawi H, Riboyad-Levin J, Klein E (1999). Chronic repetitive transcranial magnetic stimulation alters beta-adrenergic and 5-HT2 receptor characteristics in rat brain. Brain Res.

[74] Baeken C, De Raedt R, Bossuyt A, Van Hove C, Mertens J, Dobbeleir A (2011). The impact of HF-rTMS treatment on serotonin(2A) receptors in unipolar melancholic depression. Brain Stimul.

[75] Malaguti A, Rossini D, Lucca A, Magri L, Lorenzi C, Pirovano A (2011). Role of COMT, 5-HT(1A), and SERT genetic polymorphisms on antidepressant response to Transcranial Magnetic Stimulation. Depression anxiety.

[76] Zanardi R, Magri L, Rossini D, Malaguti A, Giordani S, Lorenzi C (2007). Role of serotonergic gene polymorphisms on response to transcranial magnetic stimulation in depression. Eur Neuropsychopharmacol.

[77] Xu Y, Kappen M, Peremans K, De Bundel D, Van Eeckhaut A, Van Laeken N (2022). Accelerated HF-rTMS Modifies SERT Availability in the Subgenual Anterior Cingulate Cortex: A Canine [11C]DASB Study on the Serotonergic System. J Clin Med.

[78] Liston C, Chen AC, Zebley BD, Drysdale AT, Gordon R, Leuchter B (2014). Default mode network mechanisms of transcranial magnetic stimulation in depression. Biol psychiatry.

[79] Vogt BA, Pandya DN (1987). Cingulate cortex of the rhesus monkey: II. Cortical afferents. J Comp Neurol.

[80] Hoogendam JM, Ramakers GM, Di Lazzaro V (2010). Physiology of repetitive transcranial magnetic stimulation of the human brain. Brain Stimul.

[81] Huang YZ, Rothwell JC, Chen RS, Lu CS, Chuang WL (2011). The theoretical model of theta burst form of repetitive transcranial magnetic stimulation. Clin Neurophysiol.

[82] Cambiaghi M, Cherchi L, Masin L, Infortuna C, Briski N, Caviasco C (2021). High-frequency repetitive transcranial magnetic stimulation enhances layer II/III morphological dendritic plasticity in mouse primary motor cortex. Behav Brain Res.

[83] Zanderigo F, Pantazatos S, Rubin-Falcone H, Ogden RT, Chhetry BT, Sullivan G (2018). In vivo relationship between serotonin 1A receptor binding and gray matter volume in the healthy brain and in major depressive disorder. Brain Struct Funct.

[84] Kraus C, Hahn A, Savli M, Kranz GS, Baldinger P, Höflich A (2012). Serotonin-1A receptor binding is positively associated with gray matter volume - a multimodal neuroimaging study combining PET and structural MRI. NeuroImage.

[85] Burnet PW, Eastwood SL, Harrison PJ (1997). [3H]WAY-100635 for 5-HT1A receptor autoradiography in human brain: a comparison with [3H]8-OH-DPAT and demonstration of increased binding in the frontal cortex in schizophrenia. Neurochem Int.

[86] Azmitia EC, Gannon PJ, Kheck NM, Whitaker-Azmitia PM (1996). Cellular localization of the 5-HT1A receptor in primate brain neurons and glial cells. Neuropsychopharmacology.

[87] Clarke D, Beros J, Bates KA, Harvey AR, Tang AD, Rodger J (2021). Low intensity repetitive magnetic stimulation reduces expression of genes related to inflammation and calcium signalling in cultured mouse cortical astrocytes. Brain Stimul.

[88] Martinot JL, Hardy P, Feline A, Huret JD, Mazoyer B, Attar-Levy D (1990). Left prefrontal glucose hypometabolism in the depressed state: a confirmation. Am J Psychiatry.

[89] Aleman A (2013). Use of repetitive transcranial magnetic stimulation for treatment in psychiatry. Clin Psychopharmacol Neurosci.

[90] Schutter DJ (2009). Antidepressant efficacy of high-frequency transcranial magnetic stimulation over the left dorsolateral prefrontal cortex in double-blind sham-controlled designs: a meta-analysis. Psychol Med.

[91] Li CT, Chen MH, Juan CH, Liu RS, Lin WC, Bai YM (2018). Effects of prefrontal theta-burst stimulation on brain function in treatment-resistant depression: A randomized sham-controlled neuroimaging study. Brain Stimul.

[92] Plewnia C, Pasqualetti P, Große S, Schlipf S, Wasserka B, Zwissler B (2014). Treatment of major depression with bilateral theta burst stimulation: a randomized controlled pilot trial. J Affect Disord.

[93] Voigt JD, Leuchter AF, Carpenter LL (2021). Theta burst stimulation for the acute treatment of major depressive disorder: a systematic review and meta-analysis. Transl Psychiatry.

[94] Lepping P, Schönfeldt-Lecuona C, Sambhi RS, Lanka SV, Lane S, Whittington R (2014). A systematic review of the clinical relevance of repetitive transcranial magnetic stimulation. Acta Psychiatr Scand.

[95] Kaster TS, Downar J, Vila-Rodriguez F, Thorpe KE, Feffer K, Noda Y (2019). Trajectories of response to dorsolateral prefrontal rTMS in major depression: a THREE-D study. The. Am J Psychiatry.

[96] Mutz J, Vipulananthan V, Carter B, Hurlemann R, Fu CHY, Young AH (2019). Comparative efficacy and acceptability of non-surgical brain stimulation for the acute treatment of major depressive episodes in adults: systematic review and network meta-analysis. BMJ.

[97] Eisenegger C, Treyer V, Fehr E, Knoch D (2008). Time-course of “off-line” prefrontal rTMS effects-a PET study. NeuroImage.

[98] Gryglewski G, Lanzenberger R, Kranz GS, Cumming P (2014). Meta-analysis of molecular imaging of serotonin transporters in major depression. J Cereb Blood Flow Metab.

[99] Moses-Kolko EL, Price JC, Thase ME, Meltzer CC, Kupfer DJ, Mathis CA (2007). Measurement of 5-HT1A receptor binding in depressed adults before and after antidepressant drug treatment using positron emission tomography and [11C]WAY-100635. Synapse.

[100] Choi YK, Gardner MP, Tarazi FI (2017). Developmental effects of antipsychotic drugs on serotonin receptor subtypes. Synapse.

[101] Haddjeri N, Blier P, de Montigny C (1998). Long-term antidepressant treatments result in a tonic activation of forebrain 5-HT1A receptors. J Neurosci.

[102] Hensler JG (2003). Regulation of 5-HT1A receptor function in brain following agonist or antidepressant administration. Life Sci.

