# Supplementary material for: Effects of bilateral sequential theta-burst stimulation on 5-HT1A receptors in the dorsolateral prefrontal cortex in treatment-resistant depression: a proof-of-concept trial
Source: Transl Psychiatry. 2023 Feb 1;13:33. doi: 10.1038/s41398-023-02319-3 (PMC9892572; doi:10.1038/s41398-023-02319-3)
Supplement: Supplementary file 1 — Supplemental material [file 41398_2023_2319_MOESM1_ESM.docx]

***Supplement***

**Effects of bilateral sequential theta-burst stimulation on 5-HT_1A_ receptors in the dorsolateral prefrontal cortex in treatment-resistant depression: a proof-of-concept trial**

*TBS-induced changes in 5-HT_1A_ receptor in TRD*

Matej Murgaš, MSc.^1,2*^, Dr. Jakob Unterholzner^1,2*^, Peter Stöhrmann, MSc.^1,2^,

Dr. Cécile Philippe^3^, Dr. Godber M. Godbersen^1,2^, Dr. Lukas Nics^3^, Murray B. Reed, MSc^1,2^,

Dr. Chrysoula Vraka^3^, Dr. Thomas Vanicek^1,2^, Prof. Wolfgang Wadsak^3^,

Prof. Georg S. Kranz^1,4^, Prof. Andreas Hahn^1,2^, Prof. Markus Mitterhauser^3,5,6^,

Prof. Marcus Hacker^3^, Prof. Siegfried Kasper^1#^, Prof. Rupert Lanzenberger^1,2#^,

Prof. Pia Baldinger-Melich^1,2^

(* contributed equally)

*^1^Department of Psychiatry and Psychotherapy, Clinical Division of General Psychiatry,*

*Medical University of Vienna, Austria*

*^2^Comprehensive Center for Clinical Neurosciences and Mental Health, Medical University of Vienna, Austria*

*^3^Department of Biomedical Imaging and Image-guided Therapy, Division of Nuclear Medicine,*

*Medical University of Vienna, Austria*

*^4^Department of Rehabilitation Sciences, The Hong Kong Polytechnic University, Hung Hom, Hong Kong*

*^5^Ludwig Boltzmann Institute Applied Diagnostics, Vienna, Austria*

*^6^Department of Chemistry, Institute of Inorganic Chemistry, University of Vienna, Austria*

| Patient ID | Diagnosis | Treatment  group | Antidepressants | | | | | | Antipsychotics | Mood-Stabilizer | Anxiolytics |
| --- | --- | --- | --- | --- | --- | --- | --- | --- | --- | --- | --- |
|  |  |  | SSRI | SNRI | NAssA | TCA | SMS | Other |  |  |  |
| 1 | Recurrent depressive disorder | Sham | sertraline | - | - | - | - | - | - | - | pregabalin |
| 2 | Recurrent depressive disorder | Bilateral TBS | escitalopram | - | - | - | - | - | quetiapine  (up to 50mg as required) | lithium | pregabalin |
| 3 | Recurrent depressive disorder | Sham | - | duloxetine | - | - | - | - | prothipendyl | lithium | lorazepam  (up to 1.25mg as required) |
| 4 | Recurrent depressive disorder | Bilateral TBS | - | milnacipran | - | melitracen | - | - | flupentixole, prothipendyl | - | alprazolam (up to 0.5mg as required) |
| 5 | Recurrent depressive disorder | Sham | - | venlafaxine | - | - | - | - | - | lamotrigine | lorazepam (up to 2mg as required), zolpidem |
| 6 | Recurrent depressive disorder | Bilateral TBS | sertraline | - | - | - | - | - | - | - | - |
| 7 | Major depressive disorder | Bilateral TBS | escitalopram | - | - | - | - | mianserine | - | - | - |
| 8 | Recurrent depressive disorder | Bilateral TBS | escitalopram | - | - | - | - | - | - | - | - |
| 9 | Recurrent depressive disorder, dysthamia | Bilateral TBS | - | venlafaxine | - | - | - | - | - | lithium | - |
| 10 | Major depressive disorder | Bilateral TBS | - | milnacipran | - | - | - | - | - | - | - |
| 11 | Recurrent depressive disorder | Bilateral TBS | - | - | - | melitracen | - | - | flupentixole | - | - |

**Supplementary table S1:** Concomitant medication of study subjects, both depressive episode and recurrent depressive disorder diagnoses were therapy-resistant
